# Pancreatic cancer mimicking ectopic pancreas origin: a rare case after neonatal pyloromyotomy

**DOI:** 10.1007/s12328-026-02352-x

**Published:** 2026-05-15

**Authors:** Yuta Hasegawa, Takazumi Tsunenari, Sho Ogata, Chikako Sato, Yoshitaka Imoto, Hiroyuki Horiguchi, Takahiro Einama, Hironori Tsujimoto, Hideki Ueno, Yoji Kishi

**Affiliations:** 1 Department of Surgery, National Defense Medical College, 3-2 Namiki, Tokorozawa, 359-8513 Saitama Japan; 2 Department of Laboratory Medicine, National Defense Medical College Hospital, 3-2 Namiki, Tokorozawa, 359-8513 Saitama Japan

**Keywords:** Ectopic pancreas, Pancreatic cancer, Pyloromyotomy

## Abstract

Pyloromyotomy is a technique for relieving infantile hypertrophic pyloric stenosis and is performed almost safely without severe complications during long-term outcomes. To the best of our knowledge, this is the first reported case of pancreatic cancer arising from the pancreatic parenchyma embedded within the pyloric ring after neonatal pyloromyotomy. A 39-year-old woman with a history of infantile hypertrophic pyloric stenosis underwent a pyloromyotomy during the neonatal period and presented with progressive nausea and vomiting. Imaging studies suggested the presence of ectopic pancreatic or gastric cancer invading the pancreas. After laparotomy, tumor invasion of the pancreatic head was identified; therefore, pancreaticoduodenectomy was performed. Pathological examination confirmed pancreatic cancer arising from the pancreatic parenchyma embedded within the duodenal and pyloric muscle layers. The patient completed 6 months of adjuvant chemotherapy with oral S-1. *P*eritoneal dissemination was observed 23 months postoperatively. Abnormal embedding of the pancreatic head in the walls of the duodenum and pyloric ring was considered to be related to neonatal pyloromyotomy. This alteration requires careful preoperative evaluation and flexible surgical planning to achieve curative resection of atypical presentations of gastrointestinal malignancies. The cancer in the present case may be associated with long-term anatomical alterations following neonatal pyloromyotomy, although a direct causal relationship cannot be established.

**Supplementary Information:**

The online version contains supplementary material available at 10.1007/s12328-026-02352-x.

## Introduction

Pyloromyotomy is usually used to treat infantile hypertrophic pyloric stenosis and was originally described by Fredet and Ramstedt [1]. The incidences of postoperative morbidity and mortality after pyloromyotomy are reportedly low [2–4]. The reported overall complication rates range 2–6%, including wound infection (2–4%), incomplete pyloromyotomy (0.3–1.2%), and mucosal perforation (1–1.7%) [5]. 

Open surgery was previously performed; however, laparoscopic procedures have recently been employed [6]. We present the case of a patient with a history of open pyloromyotomy in which the cancer was mainly located in the pyloric ring that appeared in the ectopic pancreas, but pathology confirmed that the cancer originated from abnormal embedding of the pancreatic head parenchyma in the first portion of the duodenum adjoining the pyloric cancer site. The present case may be a rare presentation of long-term outcomes following neonatal open pyloromyotomy.

### Case presentation

A 39-year-old woman presented with progressive nausea and vomiting for over 4 months, which had recently progressed to difficulty tolerating liquids. The patient underwent open pyloromyotomy for congenital hypertrophic pyloric stenosis at 20 days of age.

**Fig. 1:**
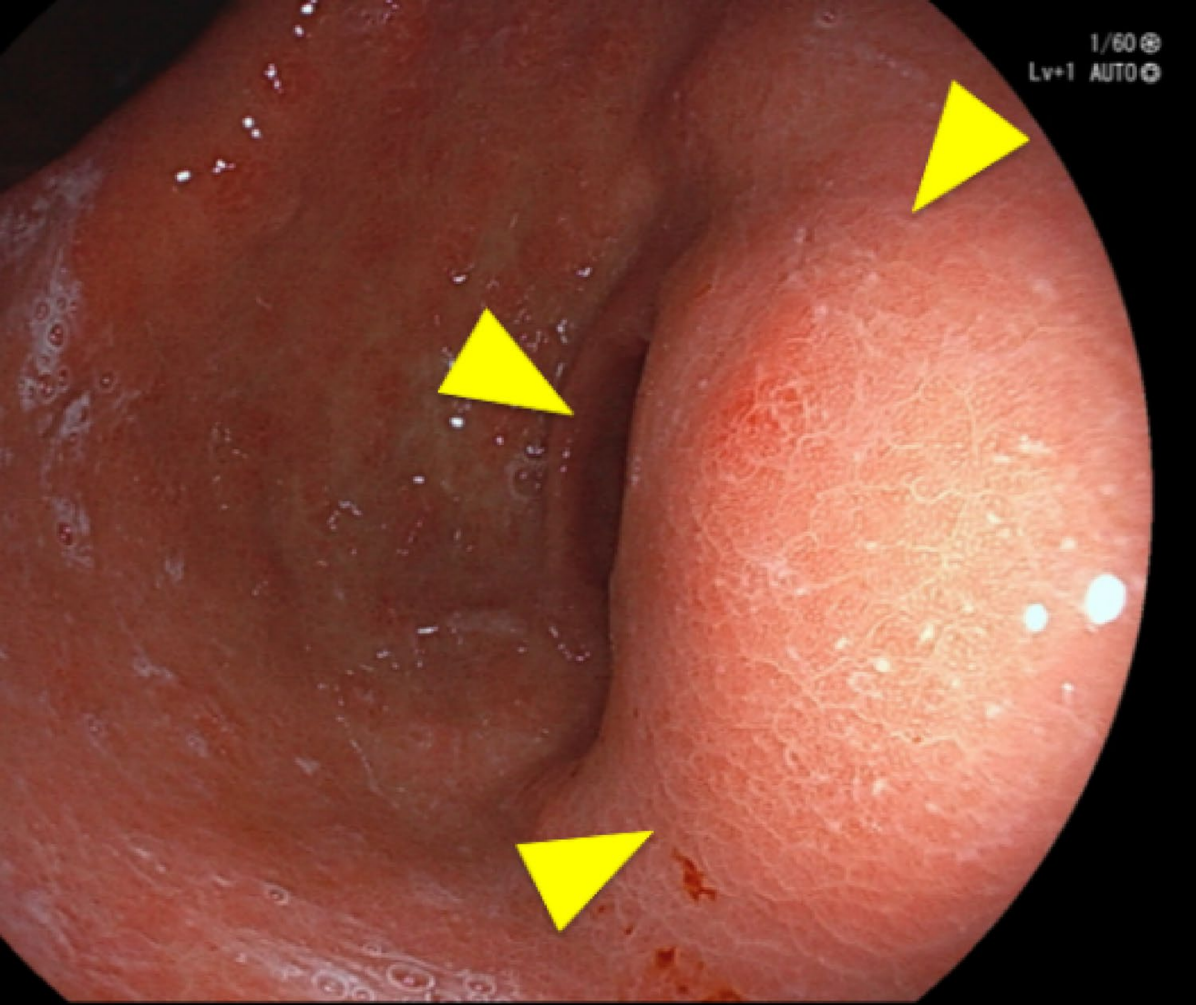
Upper gastrointestinal endoscopy depicting extrinsic compression of the pyloric region (yellow arrow) without mucosal abnormalities

**Fig. 2:**
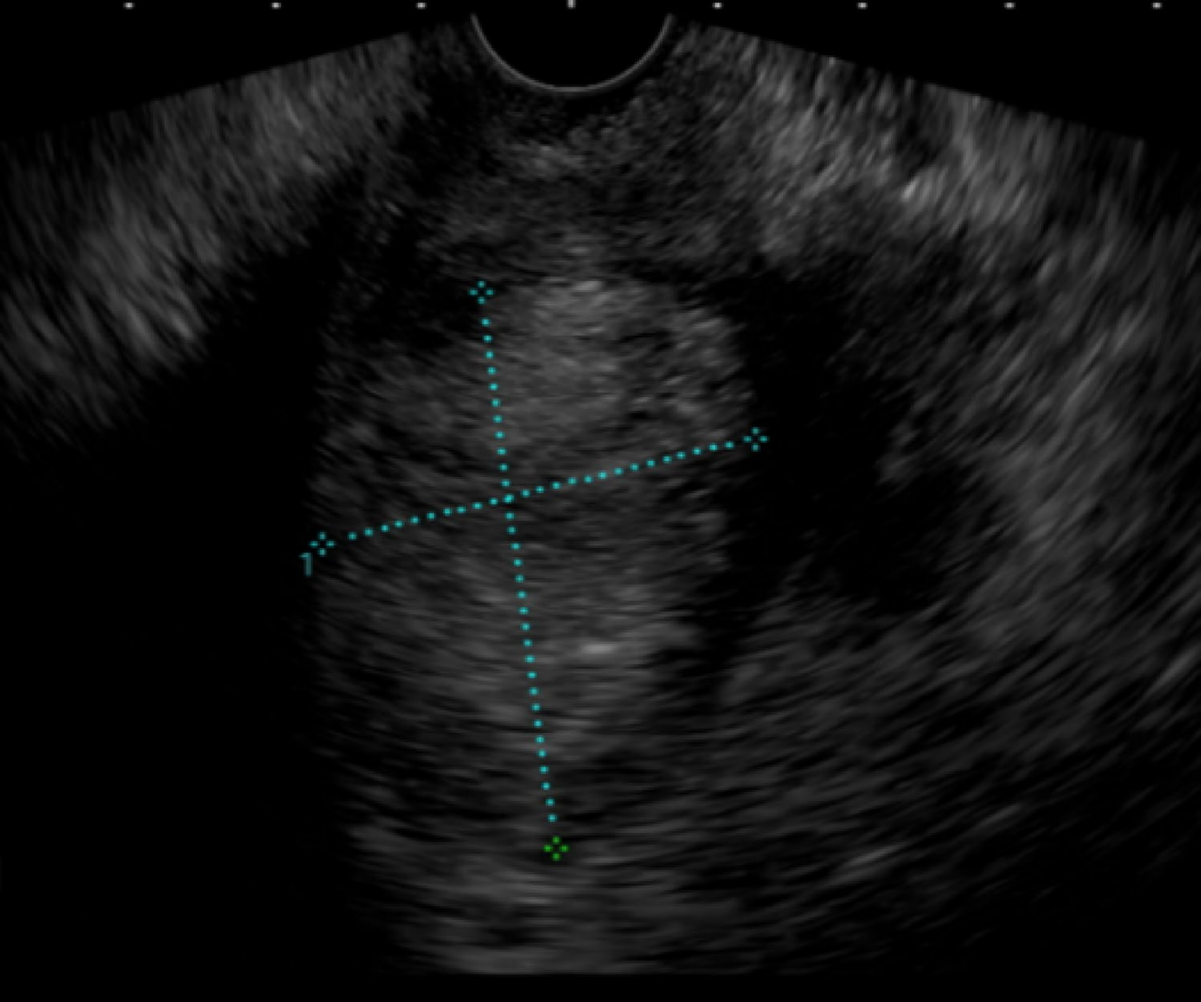
Endoscopic ultrasound-fine needle aspiration histology shows adenocarcinoma cells. Endoscopic ultrasonography image showing a 30 × 38 mm hypoechoic mass in the pyloric area

**Fig. 3:**
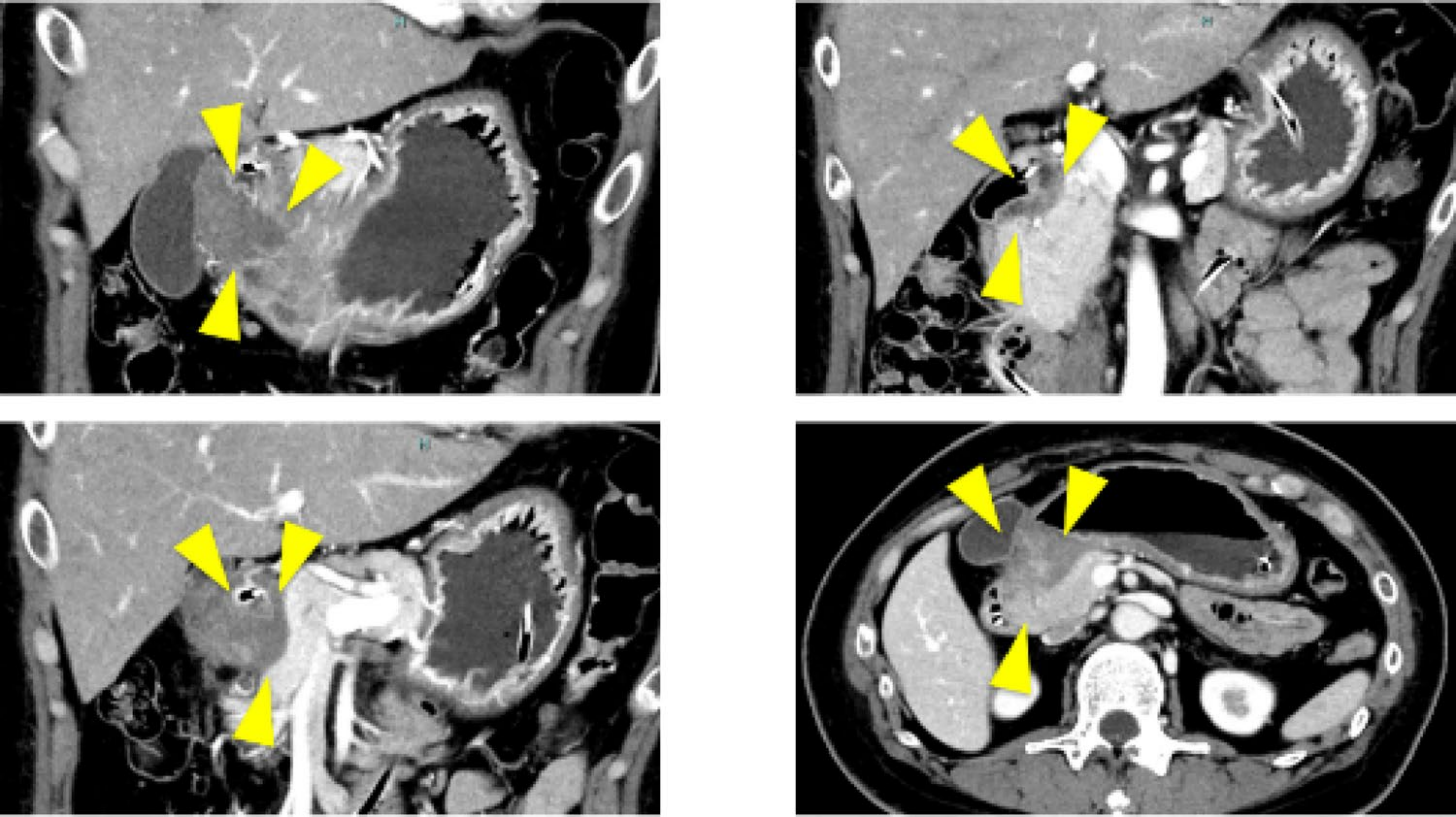
Contrast-enhanced computed tomography demonstrates circumferential thickening around the pylorus and contact with the pancreatic head

**Fig. 4:**
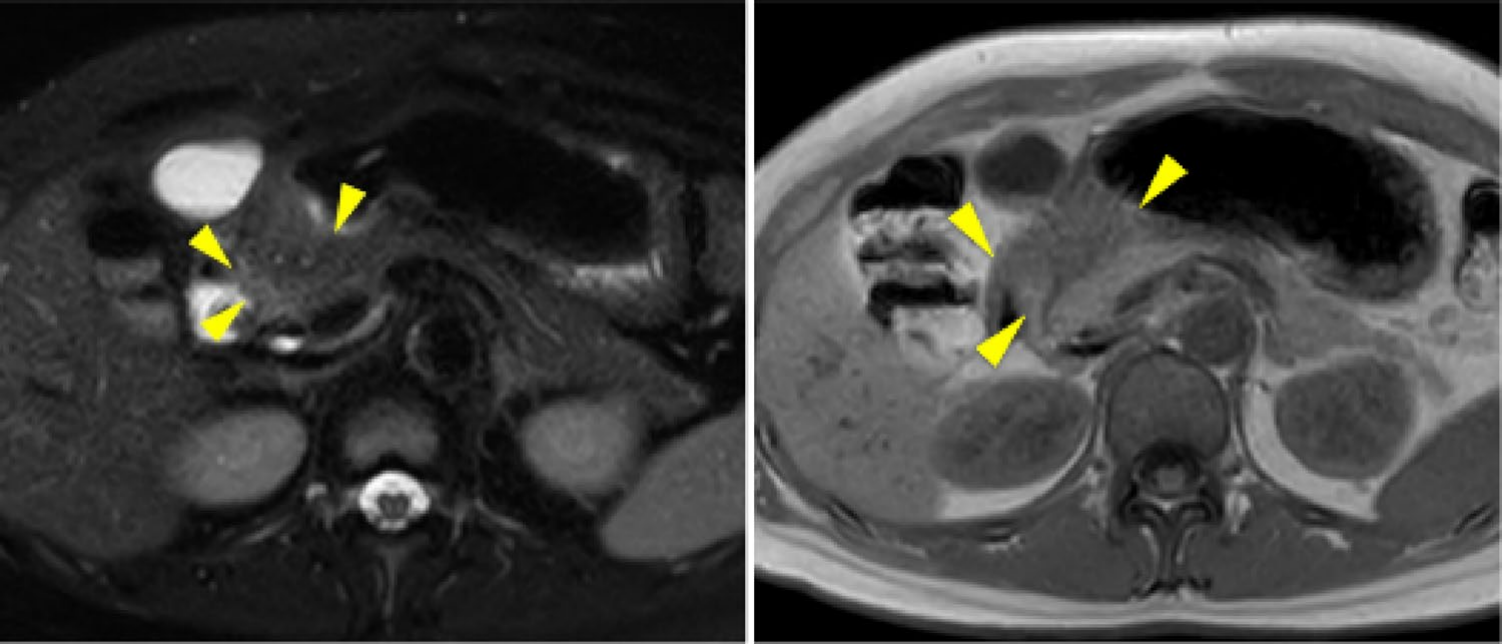
Magnetic resonance imaging (T2-weighted fat-suppressed and T1-weighted sequences) showing tubular structures broadly contacting the pancreatic parenchyma

Despite repeated outpatient evaluations and follow-up, her symptoms failed to improve, prompting presentation to the emergency department for urgent admission. The patient was hemodynamically stable except for mild tachycardia. The abdominal examination findings were unremarkable. Laboratory test results, including tumor marker levels, were within normal limits (Table 1). Abdominal radiography revealed gastric fluid levels and retention. Upper gastrointestinal endoscopy revealed extrinsic compression in the pyloric region without mucosal abnormalities (Fig. 1). Endoscopic ultrasound (EUS) revealed a 30 × 38 mm hypoechoic mass with duct-like structures and cystic components in the submucosa to the muscularis propria (Fig. 2). EUS-guided fine-needle aspiration revealed malignant epithelial cells forming irregular glandular structures, consistent with adenocarcinoma. Contrast-enhanced computed tomography (CT) and magnetic resonance imaging (MRI) revealed circumferential thickening around the pylorus with gradual enhancement toward the equilibrium phase, increased peripancreatic fat density, and broad contact with the pancreatic head (Figs. 3 and 4).

**Table 1:** Preoperative laboratory data

Parameter	Values	Unit	Parameter	Value	Unit
T-bil	0.96	mg/dL	Na	143	mmol/L
AST	13	UL	K	3.1	mmol/L
ALT	14	U/L	Cl	104	mmol/L
LD	166	U/L	CRP	0.16	mg/dL
ALP	64	U/L	WBC	6.8 × 10^3	/µL
γ-GTP	7	U/L	RBC	5.00 × 10^6	/µL
TP	6.9	g/dL	Hb	14.7	g/dL
Alb	4.4	g/dL	Ht	43.1	
Glu	115	mg/dL	PLT	214 × 10^3	/µL
Amy	76	U/L	CA19-9	11.5	U/mL
BUN	11	mg/dL	CEA	1	ng/mL
Cre	0.64	mg/dL			

The patient was scheduled for distal gastrectomy, with possible conversion to pancreaticoduodenectomy (PD). After laparotomy, tumor invasion of the anterior surface of the pancreatic head was suspected, and PD was performed. Intraoperative peritoneal lavage cytology was performed at laparotomy and showed no malignant cells. Gross pathological examination confirmed a 35 × 34 × 30 mm mass that clearly invaded the pyloric muscle layer and was attached to the anterior pancreatic surface.

**Fig. 5:**
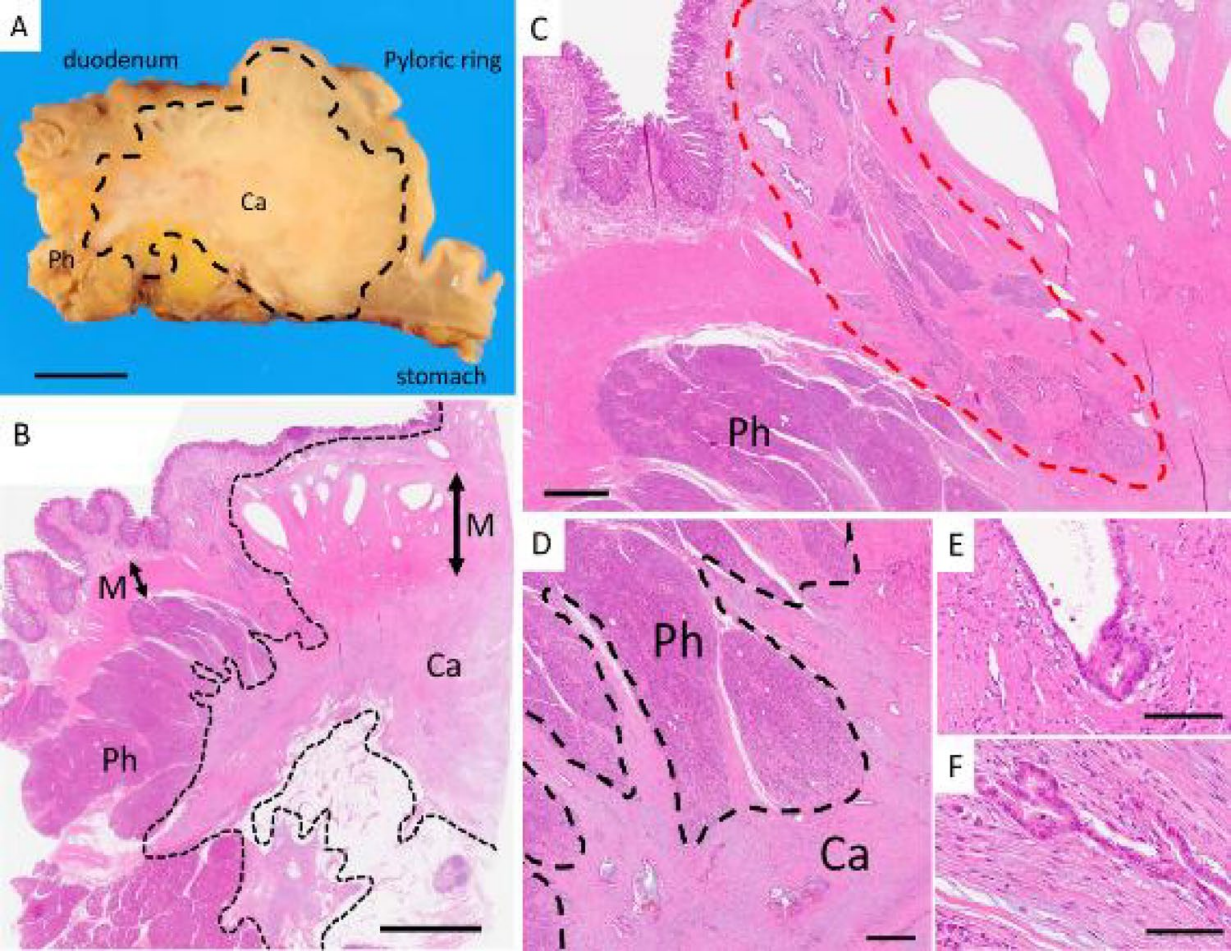
On the cut-surface, grossly, a gray- to white-colored mass (Ca in **A**, outlined by a dotted black line) is located to the pyloric ring, duodenum, and also periphery of the pancreatic head (Ph). As a background feature, non-neoplastic pancreatic head parenchyma is directly embedded in and compressing the muscularis propria of the duodenum (M in **B**, arrows), and its proximal part becomes atrophic (**C**, encircled by a dotted red line). Adjoining these structure changes, neoplastic glands proliferated mainly in the pyloric ring, and some of these glands are dilated cystically (**B** and **E**). These adenocarcinoma (Ca in **D**) are adjoining also the periphery of the pancreas head (Ph) and showing stromal desmoplastic reaction typical for pancreatic ductal adenocarcinoma (**F**). **B**–**F**: hematoxylin-eosin stain; scale bar indicates 1 cm in **A**, 5 mm in **B**, 1 mm in **C**, 500 μm in **D**, and 100 μm in **E** & **F**, respectively

Histological examination revealed a peculiar background feature in which the pancreatic head parenchyma was directly embedded in the muscularis propria of the duodenal first portion and an atrophic pancreatic component adjoining the cancer occupying the duodenum and pyloric ring (Fig. 5). Cancer cells also infiltrated and replaced the genuine pancreatic head parenchyma. It was plausible to consider its origin from the embedded pancreatic parenchyma rather than from the ectopic pancreas or gastric origin. These cancer cells were classified as moderately to well-differentiated adenocarcinomas with marked desmoplastic stroma, typical of pancreatic ductal adenocarcinoma. In addition, histological examination of the resected specimen demonstrated glandular structures and stromal reactions characteristic of pancreatic ductal origin, supporting the diagnosis of pancreatic ductal adenocarcinoma. Immunohistochemically, the tumor cells were positive for *keratin 7*,* keratin 19*,* MUC1 (luminal)*,* IMP3*,* CDX*,* the tumor cells were positive for2 (partially)*, and loss of *SMAD4* expression was not evident. The tumor was considered to be a pT3pN0M0 pStage IIA pancreatic cancer according to the staging system of the UICC/AJCC 8th edition [7, 8].

The postoperative course was uneventful, and the patient was discharged on postoperative 18th day. She completed 6 months of postoperative adjuvant S-1 therapy; however, peritoneal dissemination was confirmed 23 months after surgery.

## Discussion

In the present case, successful intraoperative conversion to PD was crucial to achieve R0 resection and to simultaneously elucidate the full histology of cancer localization and the anatomical association of the pyloric ring, duodenum, and pancreas. The present case had a history of neonatal pyloromyotomy, and it exhibited peculiar clinicopathologic characteristics as follows: (1) the tumor originated in the abnormally embedded pancreatic parenchyma into the duodenal first portion, (2) imaging studies suggested the diagnosis of gastric cancer of ectopic pancreatic origin in the gastric pyloric ring, and (3) the patient was relatively young for pancreatic cancer.

In the present case, in addition to an adenocarcinoma located at the boundary of the stomach, duodenum, and pancreas, we found abnormal embedding of noncancerous pancreatic head parenchyma into the duodenal muscularis propria. This embedding was limited to the duodenal first portion and the wall near the pancreatic head, devoid of the free duodenal wall. Although congenital anomalies in embryogenesis, such as the annular pancreas, might be considered, there was no sign of annular pancreas in the duodenal second portion in the present case, and there have been no reports of this type of pancreas embedding in the duodenal first portion. Further, subtle congenital pancreatic positional or fusion anomalies, such as an accessory pancreatic lobe, cannot be completely excluded, but congenital embedding was considered less likely in this case because neonatal pyloromyotomy relieved the symptoms of pyloric stenosis and no reintervention or reoperation had been required postoperatively. Pyloromyotomy is considered safe, without severe complications, with a rare incidence of duodenal perforation or intestinal adhesions. In the present case, it is plausible to consider that abnormal embedding may have occurred as a result of postoperative anatomical alteration following the operative procedure, although a congenital developmental variant cannot be definitively excluded. Such anatomical alterations require careful preoperative evaluation and timely intraoperative decision-making, which are crucial for achieving curative resection in these rare but clinically significant scenarios. No definitive histological evidence of surgical scarring was identified; therefore, the proposed mechanism remains hypothetical.

Clinically, lesions arising from an ectopic pancreas often mimic gastric or duodenal tumors on endoscopy, EUS, CT, and MRI, complicating the preoperative diagnosis. Radiologically, carcinomas originating from ectopic pancreas in the pylorus typically present as mural thickening with gradual enhancement on contrast-enhanced CT, consistent with a submucosal-muscularis origin [9, 10]. Endoscopically, the overlying mucosa is generally preserved, leading to frequent misidentification of submucosal tumors. On EUS, these lesions appear as heterogeneous hypoechoic masses arising from the third to fourth layers, occasionally with residual pancreatic acini or duct-like structures, indicative of malignant transformation [10, 11]. In our patient, circumferential pyloric wall thickening with gradual enhancement, preserved overlying mucosa, and a heterogeneous hypoechoic mass arising from the third and fourth layers on EUS closely resembled these typical features, leading to the preoperative diagnosis of carcinoma arising from an ectopic pancreas in the pylorus. Although the patient had a history of neonatal pyloromyotomy, postoperative anatomical distortion was not strongly suspected preoperatively because of the long asymptomatic interval and the imaging features closely resembling those reported for carcinoma arising from ectopic pancreas, including a submucosal lesion with preserved overlying mucosa, gradual enhancement on contrast-enhanced CT, and a heterogeneous hypoechoic mass arising from the third to fourth layers with duct-like or cystic structures on EUS [10, 12].

In the present case, the preoperative differential diagnoses included ectopic pancreatic carcinoma and gastric cancer invading the pancreas. Ectopic pancreas is observed in 0.5–15% of autopsy cases [13, 14] and is more common in males, with the stomach (particularly the prepyloric region) and duodenum being the most frequent sites [13]. Although most ectopic pancreatic tumors are asymptomatic, malignant transformation is rare. The reported cancers are typically adenocarcinomas occurring in patients in their sixth or seventh decade of life, with average tumor sizes of 27–48 mm and moderate to well differentiation [15]. In contrast to previous reports of carcinoma arising in ectopic pancreas (Table 2), pathological examination in the present case demonstrated typical features of pancreatic ductal adenocarcinoma and its anatomical continuity between the abnormally embedded pancreatic tissue and the underlying genuine pancreas head parenchyma, definitively excluding “true ectopia” origin because ectopia should be apart from the main organ. To our knowledge, pancreatic cancer arising from the pancreatic tissue embedded within the pyloric ring after neonatal pyloromyotomy has not been previously documented, underscoring the novelty of the present case.

**Table 2:** Comparison of characteristics between carcinoma in ectopic pancreas and the present case

Feature	Carcinoma in ectopic pancreas (previous reports [9–14])	Embedded pancreas cancer (present case)
Age at diagnosis	Usually 50–70 s	39 years
Location	Gastric antrum/pylorus or duodenum (submucosal)	Pyloric ring/first duodenum with pancreatic head continuity
Relationship to main pancreas	No anatomical continuity with the main pancreas	Clear continuity with pancreatic head parenchyma
Endoscopic appearance	Submucosal tumor-like lesion with normal overlying mucosa	Extrinsic compression, intact mucosa
EUS findings	Submucosal hypoechoic mass, sometimes duct-like structures	Hypoechoic mass with duct-like structures, broad contact with pancreatic head
CT / MRI	Mural thickening, gradual enhancement	Circumferential pyloric thickening with broad contact with pancreatic head

In the present case, the patient had pancreatic cancer in her fourth decade of life, relatively young. Previous studies have suggested that prior gastric surgery may increase pancreatic cancer risk by 1.8–3.6-fold via persistent inflammation and exposure to growth factors such as interleukin-6, tumor necrosis factor-α, and epidermal growth factor [16]. Additionally, alterations in incretin hormone levels after surgery may stimulate pancreatic cell proliferation [17–19]. Embedded pancreatic tissue chronically exposed to gastric acid and enzymes may theoretically develop persistent inflammation and fibrosis, potentially contributing to neoplastic transformation [20, 21]. However, in the present case, although focal atrophy of the embedded non-neoplastic pancreatic parenchyma was observed, overt histological features of chronic pancreatitis, such as marked fibrosis, were limited, and this hypothesis remains speculative. In contrast, there are no negative functional effects on the digestive tract in long-term outcomes [22], and the pathological features of hypertrophic pyloric stenosis have been resolved in the short term after pyloromyotomy [23]. However, rapid gastric emptying or bile regurgitation can occur in diseased and postoperative pylorus due to decreased sphincter function. These are the potential risks for malignancy. In the present case, the duodenal mobility should have decreased due to the relative loss of muscular volume by the abnormally embedded pancreas. The cancer in the present case may be associated with long-term anatomical alterations following neonatal pyloromyotomy; however, a direct causal relationship cannot be established. Therefore, the proposed mechanism would not be definitive.

Here, we report an extremely rare case of pancreatic cancer arising from the pancreatic parenchyma embedded within the pyloric ring after neonatal pyloromyotomy. The cancer mimicked a pancreatic cancer of ectopic origin. Operative procedures at a young age may change the anatomical location, which is sometimes difficult to understand.

## Supplementary Information

Below is the link to the electronic supplementary material.

Supplementary Material 1

Supplementary Material 2

## Data Availability

Data used in this study are available from the corresponding author upon request.

## Abbreviations

PD: Pancreaticoduodenectomy
CT: Computed tomography
MRI: Magnetic resonance imaging
IMP3: Insulin-like growth factor 2 messenger RNA-binding protein 3
CDX2: Caudal-type homeobox protein 2
SMAD4: SMAD family member 4
