# Supplementary material for: Pancreatic cancer mimicking ectopic pancreas origin: a rare case after neonatal pyloromyotomy
Source: Clin J Gastroenterol. 2026 May 15;19(4):801–7. doi: 10.1007/s12328-026-02352-x (PMC13424258; doi:10.1007/s12328-026-02352-x)
Supplement: Supplementary file 1 — Supplementary Material 1 [file 12328_2026_2352_MOESM1_ESM.pdf]

Journal Name: ☐ Journal of Gastroenterology／☒ Clinical Journal of Gastroenterology

The manuscript entitled:

Pancreatic cancer mimicking ectopic pancreas origin: a rare case postneonatal pyloromyotomy

is original. The author(s) hereby certifies(-fy) that none of the material in this manuscript has been or will be published and none is currently under consideration for publication elsewhere, and that the Conflict of Interest Disclosure Statement on Editorial Manager® was completed at the time of submission.

Date	Full name (printed)	Signature
2026.1.7	Yuta Hasegawa	Yuta Hasegawa
2026.1.8	Takazumi Tsunenari	T. Tsunenari
2026.1.8	Sho Ogata	[illegible]
2026.1.8	Chikako Sato	Chikako Sato
2026.1.8	Yoshitaka Inoto	Yoshitaka Inoto
2026.1.8	Hiroyuki Horiguchi	K. Horiguchi
2026.1.8	Takahiro Einama	T. Einama
2026.1.8	HIDEKI UENO	Hideki Ueno
Jan.7, 2026	Yoji Kishi	[illegible]

The certificate must be signed by all authors.

【PLEASE NOTE】

Please provide **DIRECT HANDWRITTEN** signatures by all authors.
Signature by proxy, digital signature or pasting image of the signature are **NOT ACCEPTABLE**.

Journal Name: ☐ **Journal of Gastroenterology** / ☒ **Clinical Journal of Gastroenterology**

The manuscript entitled:

Pancreatic cancer mimicking ectopic pancreas origin: a rare case postneonatal pyloromyotomy

is original. The author(s) hereby certifies(-fy) that none of the material in this manuscript has been or will be published and none is currently under consideration for publication elsewhere, and that the Conflict of Interest Disclosure Statement on Editorial Manager® was completed at the time of submission.

Date Jan. 6. 2015 Full name (printed) Hironori Tsujimoto Signature [illegible]

Date .... Full name (printed) .... Signature ....

Date .... Full name (printed) .... Signature ....

Date .... Full name (printed) .... Signature ....

Date .... Full name (printed) .... Signature ....

Date .... Full name (printed) .... Signature ....

Date .... Full name (printed) .... Signature ....

Date .... Full name (printed) .... Signature ....

Date .... Full name (printed) .... Signature ....

Date .... Full name (printed) .... Signature ....

Date .... Full name (printed) .... Signature ....

Date .... Full name (printed) .... Signature ....

Date .... Full name (printed) .... Signature ....

Date .... Full name (printed) .... Signature ....

Date .... Full name (printed) .... Signature ....

The certificate must be signed by all authors.

【PLEASE NOTE】

Please provide **DIRECT HANDWRITTEN** signatures by all authors.
Signature by proxy, digital signature or pasting image of the signature are **NOT ACCEPTABLE**.
