# Supplementary material for: Pancreatic cancer mimicking ectopic pancreas origin: a rare case after neonatal pyloromyotomy
Source: Clin J Gastroenterol. 2026 May 15;19(4):801–7. doi: 10.1007/s12328-026-02352-x (PMC13424258; doi:10.1007/s12328-026-02352-x)
Supplement: Supplementary file 2 — Supplementary Material 2 [file 12328_2026_2352_MOESM2_ESM.pdf]

Certificate of Exclusive Submission and ICMJE DISCLOSURE FORM

Date: 2026.1.13
Your Name: Yuta Hasegawa

Manuscript Title: Pancreatic cancer mimicking ectopic pancreas origin: a rare case postneonatal pyloromyotomy

Manuscript number (if known):

Redundant or Duplicate Publication

When submitting a paper, an author should always make a full statement to the editor about all submissions and previous reports that might be regarded as redundant or duplicate publication of the same or very similar work. The author should alert the editor if the work includes subjects about whom a previous report has been published. Any such work should be referred to and referenced in the new paper. Copies of such material should be included with the submitted paper to help the editor decide how to deal with the matter.
If redundant or duplicate publication is attempted or occurs without such notification, authors should expect editorial action to be taken. At the least, prompt rejection of the submitted manuscript should be expected. If the editor was not aware of the violations and the article has already been published, then a notice of redundant or duplicate publication will probably be published with or without the author's explanation or approval.

Acceptable Secondary Publication

Secondary publication in the same or another language, especially in other countries, is justifiable, and can be beneficial, provided all of the following conditions are met:

- The authors have received approval from the editors of both journals; the editor concerned with secondary publication must have a photocopy, reprint, or manuscript of the primary version.
- The priority of the primary publication is respected by a publication interval of at least one week (unless specifically negotiated otherwise by both editors).
- The paper for secondary publication is intended for a different group of readers; an abbreviated version could be sufficient.
- The secondary version reflects faithfully the data and interpretations of the primary version.
- A footnote on the title page of the secondary version informs readers, peers, and documenting agencies that the paper has been published in whole or in part and states the primary reference. A suitable footnote might read: "This article is based on a study first reported in the [title of journal, with full reference]."

Conflict of Interest

In the interest of transparency, we ask you to disclose all relationships/activities/interests listed below that are related to the content of your manuscript. "Related" means any relation with for-profit or not-for-profit third parties whose interests may be affected by the content of the manuscript. Disclosure represents a commitment to transparency and does not necessarily indicate a bias. If you are in doubt about whether to list a relationship/activity/interest, it is preferable that you do so.

The following questions apply to the author's relationships/activities/interests as they relate to the current manuscript only.

The author's relationships/activities/interests should be defined broadly. For example, if your manuscript pertains to the epidemiology of hypertension, you should declare all relationships with manufacturers of antihypertensive medication, even if that medication is not mentioned in the manuscript.

In item #1 below, report all support for the work reported in this manuscript without time limit. For all other items, the time frame for disclosure is the past 36 months.

Reproduced from: International Committee of Medical Journal Editors. Uniform Requirements for Manuscripts Submitted to Biomedical Journals.
http://www.icmje.org/

Authors must ensure that all information is visible on the final PDF file they submit, as the text entered on the PDF form may not be fully reproduced when printed on paper.

		Name all entities with whom you have this relationship or indicate none (add rows as needed)	Specifications/Comments (e.g., if payments were made to you or to your institution)
**Time frame: Since the initial planning of the work**			
1	All support for the present manuscript (e.g., funding, provision of study materials, medical writing, article processing charges, etc.)<br>**No time limit for this item.**	☒None	
**Time frame: past 36 months**			
2	Grants or contracts from any entity (if not indicated in item #1 above).	☒None	
3	Royalties or licenses	☒None	
4	Consulting fees	☒None	
5	Payment or honoraria for lectures, presentations, speakers bureaus, manuscript writing or educational events	☒None	
6	Payment for expert testimony	☒None	
7	Support for attending meetings and/or travel	☒None	
8	Patents planned, issued or pending	☒None	

9	Participation on a Data Safety Monitoring Board or Advisory Board	☒ None	
10	Leadership or fiduciary role in other board, society, committee or advocacy group, paid or unpaid	☒ None	
11	Stock or stock options	☒ None	
12	Receipt of equipment, materials, drugs, medical writing, gifts or other services	☒ None	
13	Other financial or non-financial interests	☒ None	

Whether conflict of interest exists or not, the disclosure statement should be inserted in the manuscript before the References section by using the following examples for each author.

- If any of the above items (1 to 13) apply to author(s) of the article, the corresponding author should provide a statement for each author in your manuscript.
  Example:
  "A (author name) received a research grant from Z; B serves as a consultant to Y (entity name); C received lecture fees from X; D received honoraria for writing promotional material for W; E holds a patent on V; F's spouse is chairman of U."
- If none of the authors have a relationship matching the above listed items (1 to 13), please provide the following statement in your manuscript.
  Example:
  "The authors declare that they have no conflict of interest."

Please place an "X" next to the following statement to indicate your agreement:

☒ **I certify that I have answered every question and have not altered the wording of any of the questions on this form.**

Journal Name:☐Journal of Gastroenterology／☒Clinical Journal of Gastroenterology

The manuscript entitled:

Pancreatic cancer mimicking ectopic pancreas origin: a rare case postneonatal pyloromyotomy

is original. The author(s) hereby certifies(-fy) that none of the material in this manuscript has been or will be published and none is currently under consideration for publication elsewhere, and that the Conflict of Interest Disclosure Statement on Editorial Manager® was completed at the time of submission.

Date .... Full name (printed) .... Signature ....

Date .... Full name (printed) .... Signature ....

Date .... Full name (printed) .... Signature ....

Date .... Full name (printed) .... Signature ....

Date .... Full name (printed) .... Signature ....

Date .... Full name (printed) .... Signature ....

Date .... Full name (printed) .... Signature ....

Date .... Full name (printed) .... Signature ....

Date .... Full name (printed) .... Signature ....

Date .... Full name (printed) .... Signature ....

Date .... Full name (printed) .... Signature ....

Date .... Full name (printed) .... Signature ....

Date .... Full name (printed) .... Signature ....

Date .... Full name (printed) .... Signature ....

Date .... Full name (printed) .... Signature ....

The certificate must be signed by all authors.

【PLEASE NOTE】

Please provide **DIRECT HANDWRITTEN** signatures by all authors.
Signature by proxy, digital signature or pasting image of the signature are **NOT ACCEPTABLE**.
